# Zinc/Cerium-Substituted Magnetite Nanoparticles for Biomedical Applications

**DOI:** 10.3390/ijms24076249

**Published:** 2023-03-26

**Authors:** Cristina Chircov, Maria-Andreea Mincă, Andreea Bianca Serban, Alexandra Cătălina Bîrcă, Georgiana Dolete, Vladimir-Lucian Ene, Ecaterina Andronescu, Alina-Maria Holban

**Affiliations:** 1 Department of Science and Engineering of Oxide Materials and Nanomaterials, University Politehnica of Bucharest, 011061 Bucharest, Romania; 2 National Research Center for Micro and Nanomaterials, University Politehnica of Bucharest, 060042 Bucharest, Romania; 3 Faculty of Medical Engineering, University Politehnica of Bucharest, 060042 Bucharest, Romania; 4 Extreme Light Infrastructure-Nuclear Physics (ELI-NP), Horia Hulubei National R&D Institute for Physics and Nuclear Engineering, Reactorului Street No. 30, 077125 Magurele, Romania; 5 Doctoral School in Engineering and Applications of Lasers and Accelerators, University Politehnica of Bucharest, 060042 Bucharest, Romania; 6 Academy of Romanian Scientists, 54 Spl. Independentei, 050045 Bucharest, Romania; 7 Microbiology and Immunology Department, Faculty of Biology, Research Institute of the University of Bucharest, University of Bucharest, 060101 Bucharest, Romania

**Keywords:** magnetite nanoparticles, zinc, cerium, antimicrobial, anticancer, antioxidant

## Abstract

Numerous studies have reported the possibility of enhancing the properties of materials by incorporating foreign elements within their crystal lattice. In this context, while magnetite has widely known properties that have been used for various biomedical applications, the introduction of other metals within its structure could prospectively enhance its effectiveness. Specifically, zinc and cerium have demonstrated their biomedical potential through significant antioxidant, anticancer, and antimicrobial features. Therefore, the aim of the present study was to develop a series of zinc and/or cerium-substituted magnetite nanoparticles that could further be used in the medical sector. The nanostructures were synthesized through the co-precipitation method and their morpho-structural characteristics were evaluated through X-ray diffraction (XRD), inductively coupled plasma mass spectrometry (ICP-MS), X-ray photoelectron spectroscopy (XPS), dynamic light scattering (DLS), zeta potential, scanning electron microscopy (SEM), and energy dispersive X-ray spectroscopy (EDX) analyses. Furthermore, the nanostructures were subjected to a ROS-Glo H_2_O_2_ assay for assessing their antioxidant potential, MTT assay for determining their anticancer effects, and antimicrobial testing against *S. aureus*, *P. aeruginosa*, and *C. albicans* strains. Results have proven promising for future biomedical applications, as the nanostructures inhibit oxidative stress in normal cells, with between two- and three-fold reduction and cell proliferation in tumor cells; a two-fold decrease in cell viability and microbial growth; an inhibition zone diameter of 4–6 mm and minimum inhibitory concentration (MIC) of 1–2 mg/mL.

## 1. Introduction

Iron oxides are a group of naturally occurring materials with immense potential within the biomedical area owing to the iron component, which is an essential element within the organism [1]. Among them, magnetite nanoparticles are the first generation of clinically approved nanomaterials that have been intensively used for their superparamagnetic properties in novel and efficient biomedical applications [1,2,3,4,5,6,7,8,9]. Besides the possibility to adsorb and immobilize large quantities of bioactive compounds [1], magnetite nanoparticles are widely known for their intrinsic antioxidant [10], antimicrobial [11], and anticancer [12] properties, which can be potentiated through hyperthermia effects [11].

Nevertheless, the effectiveness of magnetite nanoparticles for drug delivery applications can also be improved by the intentional insertion of foreign elements within a crystal lattice to modify the properties of the starting material [13,14,15,16,17]. In this manner, the inclusion of various metal ions could further enhance the antioxidant, antimicrobial, and anticancer properties of magnetite nanoparticles [13,18].

Zinc is one of the essential microelements, as it plays fundamental roles in multiple biochemical, cellular, and physiological processes, such as cell development, proliferation, and growth, cell structure and membrane stabilization, DNA and RNA synthesis and repair, transcription gene stimulation, oxidation process delaying, enzyme activation/inhibition, immune system optimization, and antioxidant behavior [19,20,21,22,23]. While zinc ion concentrations are physiologically regulated through homeostasis to prevent excess cell uptake and toxicity, above optimum levels allow cell entry and cytotoxicity towards prokaryotes [20]. In this context, zinc can be used as an efficient inhibitor of bacterial and fungal strain growth [20,24,25]. Furthermore, numerous studies have reported the tumor suppressor effects of zinc, which generally involve the alteration of bioenergetic and metabolic mechanisms, inhibition of cell cycle activity and, consequently, of malignant advancement to metastasis and promotion of apoptosis [26,27].

Cerium is a rare earth metal with two oxidation states, i.e., Ce^3+^ and Ce^4+^, that can be converted to each other, thus ensuring its widely known antioxidant properties [28,29,30]. Hence, cerium has been applied therapeutically for the treatment of various reactive oxygen species-related disorders, such as cardiovascular diseases, inflammation, Alzheimer’s disease, and cancer [31]. In the case of cancer, cerium has been demonstrated to generate reactive oxygen species in acidic environments characteristic for tumor cells by switching from Ce^3+^ to Ce^4+^ and scavenge them in normal pH levels (non-cancerous cells) through the conversion from Ce^4+^ to Ce^3+^ [28]. Therefore, cerium-based materials can induce apoptosis in cancer cells, while protecting the surrounding normal tissues [28,30]. Additionally, cerium ions have also demonstrated bacteriostatic properties, which paved the way towards their incorporation within formulations for the treatment of skin diseases, such as wounds, burns, eczema, gangrene, intertrigo, and decubitus [31].

In this manner, incorporating zinc and/or cerium ions within the structure of magnetite nanoparticles could enhance its biomedical properties in terms of antioxidant, anticancer, and antimicrobial potential [24,32]. Nonetheless, zinc/cerium-substituted magnetite nanoparticle-based studies are considerably limited, especially within the biomedical field. Additionally, the available literature generally focuses towards one of the three directions, i.e., oxidative stress reduction, tumor treatment, and antimicrobial therapy.

Therefore, the aim of the present study was to develop a series of zinc and/or cerium-substituted magnetite nanoparticles at two different concentrations and to demonstrate the enhancement of the nanostructure potential in terms of antioxidant, anticancer, and antimicrobial activity through morpho-structural and biological characterizations.

## 2. Results

The present study aimed to obtain a series of zinc- and/or cerium-substituted magnetite nanoparticles for biomedical applications. The results of the morpho-structural characterization and biological evaluations are described in the following paragraphs.

### 2.1. X-ray Diffraction (XRD) Coupled with Rietveld Refinement

**Figure 1:**
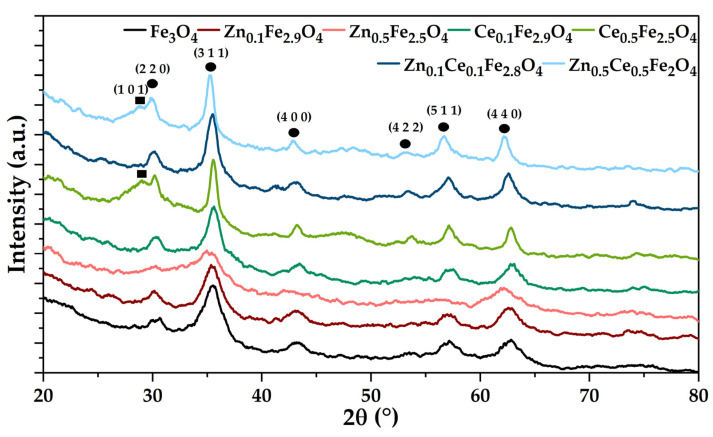
Diffractograms for magnetite and zinc/cerium-substituted nanoparticles (●—magnetite; ■—cerium oxide).

The XRD analysis coupled with Rietveld refinement was employed to qualitatively and quantitatively determine the mineral phases present within the obtained samples (Figure 1; Table 1). The diffractograms reveal the formation of magnetite in the *Fd-3m* cubic crystal system as the single mineral phase within samples Fe_3_O_4_, Zn_0.1_Fe_2.9_O_4_, and Ce_0.1_Fe_2.9_O_4_, (according to JCPDS 01-084-2782 [33,34]). By contrast, the increase in the substitution degree to 16.66% leads to the occurrence of secondary mineral phases, namely ZnO and Ce_2_O_3_ in the hexagonal crystal system (according to JCPDS 00-005-0664 [35] and JCPDS 00-023-1048 [36], respectively). Additionally, higher substitution degrees lead to the shift of the Fe_3_O_4_ main diffraction peak at lower values, especially for the Zn_0.5_Ce_0.5_Fe_2_O_4_ sample, thus confirming the presence of zinc/cerium within the lattice structure of magnetite due to larger atomic radii of zinc and cerium [37,38,39].

Furthermore, the Rietveld refinement demonstrated the formation of a significantly lower percentage of the secondary mineral phase as compared to the stoichiometrically calculated amount that would be obtained at a substitution rate of 0%. In this context, it could be assumed that the substitution yield is the highest for the co-substituted samples. Moreover, the substitution of the iron ions with zinc and/or cerium is also demonstrated through the dimensions of the unit cell parameters and the average crystallite size. Specifically, when (co-)adding zinc, the size of the unit cell increases proportionally, but the crystallite size significantly decreases at the higher concentration; in the case of cerium, the effects are the opposite, most likely due to the formation of vacancies within the unit cell. In terms of the nanoparticle crystallinity, it seems that the inclusion of the zinc and cerium ions results in the deformation of the magnetite unit cell and consequently to a decrease in crystallinity. However, the Zn_0.5_Fe_2.5_O_4_ sample seems to have a higher crystallinity than the Fe_3_O_4_ sample, which could be attributed to the presence of a secondary zinc oxide phase that is highly crystalline [40].

### 2.2. Inductively Coupled Plasma Mass Spectrometry (ICP-MS)

Subsequently, samples were subjected to ICP-MS analysis to quantify the contents of the iron, zinc, and cerium ions to further estimate the stoichiometry of the nanosystems (Table 2). It can be observed that for all samples, the amount of zinc and cerium determined through ICP-MS are similar to those added in the synthesis step. However, the iron amount is lower, which could be caused by the formation of the secondary phases. In this context, considering both the mass% of the secondary phases formed and the amounts of each ion present, the stoichiometry of the nanosystems were estimated in Table 3. Additionally, for the samples obtained at lower substitution degrees, the 5 wt% limit of detection of the diffractometer was considered as the maximum %mass of the secondary phase. Generally, the estimated stoichiometries are similar to the theoretical ones, thus indicating the successful incorporation of the zinc and/or cerium ions within the structure of magnetite.

### 2.3. X-ray Photoelectron Spectroscopy (XPS)

**Figure 2:**
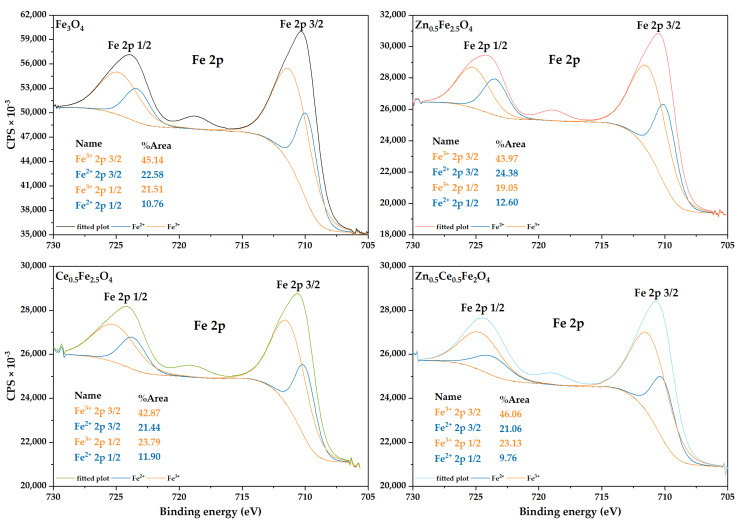
The high resolution XPS spectra and the deconvoluted signals for Fe^3+^ and Fe^2+^ of the Fe 2p.

The XPS analysis was used for assessing the Fe^2+^:Fe^3+^ ratio in order to determine the amount of zinc and/or cerium incorporated within the unit cell of magnetite and their affinity to substitute either the Fe^2+^ or the Fe^3+^ ions (Figure 2). The Fe_3_O_4_ sample is characterized by a 1:2 ratio of the Fe^2+^:Fe^3+^ ions, thus proving the formation of magnetite as the unique mineral phase. In the case of the Zn_0.5_Fe_2.5_O_4_ sample, the Fe^2+^:Fe^3+^ ratio is 1:1.7, which demonstrates a higher affinity of zinc to substitute the Fe^3+^ ion. Since the 1:2 molar ratio is maintained within the Ce_0.5_Fe_2.5_O_4_ sample, it could be assumed that the cerium substitution did not occur. However, there are many literature studies reporting several possibilities. First, Ce^3+^ is oxidized to Ce^4+^ through the reduction of Fe^3+^ to Fe^2+^, followed by the replacement of an octahedral Fe^3+^ ion and, subsequently, the reducing of another Fe^3+^ ion to Fe^2+^ to maintain charge neutrality [38]. However, this mechanism would increase the Fe^2+^ content, which is not the case for this sample. Second, another possibility involves the same initial oxidation of Ce^3+^ to Ce^4+^ and the replacement of an Fe^2+^ ion with the Ce^4+^ ion; nonetheless, the 1:2 ratio would not be maintained. Third, Ce^3+^ could replace the Fe^3+^ ion, which would also increase the Fe^2+^ amount. Considering the Zn_0.5_Ce_0.5_Fe_3_O_4_ sample, the Fe^2+^:Fe^3+^ ratio is 1:2.25. Since zinc tends to replace the Fe^3+^ ions, it could be assumed that the increase in Fe^3+^ is caused by the replacement of the Fe^2+^ with Ce^4+^. In this context, it could further be concluded that in the case of the Ce_0.5_Fe_2.5_O_4_ sample, there is a balance between the substitution of Fe^2+^ and the oxidation–reduction reaction of Ce^3+^/Ce^4+^, which maintains the 1:2 ratio.

### 2.4. Dynamic Light Scattering (DLS) and Zeta Potential

**Figure 3:**
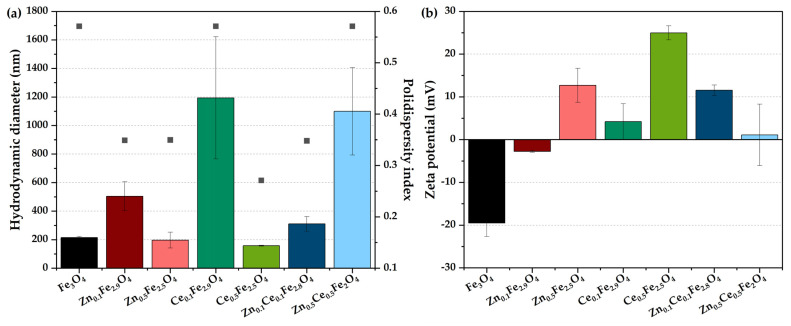
Hydrodynamic diameter (values shown as columns; expressed as mean ± SD, n = 3) and polydispersity index (values shown as points) (**a**) and zeta potential (**b**) for the zinc/cerium-substituted magnetite nanoparticles.

The subsequent characterization step consisted of assessing the stability of zinc/cerium-substituted nanostructures in terms of the agglomeration tendency in the suspension through a hydrodynamic diameter correlated with polydispersity index (Figure 3a) and zeta potential (Figure 3b) measurements. Results demonstrate that lower mono-substitution degrees increase the hydrodynamic diameter, with the highest value registered for the cerium-containing sample. Increasing the substitution degree leads to considerably reduced hydrodynamic diameters and more monodispersed size distributions [1,41]. However, the Ce_0.5_Zn_0.5_Fe_2_O_4_ sample behaves oppositely, probably caused by the heterogenous substitution with the two types of ions. Nonetheless, the high error bar calculated for the Ce_0.1_Fe_2.9_O_4_ and Ce_0.5_Zn_0.5_Fe_2_O_4_ samples could be attributed to polydisperse nanoparticles due to a non-homogenous substitution. All polydispersity index values are in the range of 0.25–0.6, which are values generally accepted for nanoparticles [42]. Since the zeta potential reflects the overall nanoparticle surface charge, it is generally regarded as a reference for the stability of the nanoparticle dispersions, i.e., values between -25 and 25 mV indicate an increased tendency of the nanoparticles to form aggregates [11,43,44,45,46]. Therefore, it can be observed that the Ce_0.5_Fe_2.5_O_4_ sample is the most stable, with a zeta potential value of +25 mV, which confirms the previous observations regarding the higher stability of the cerium-containing samples. Additionally, samples obtained at lower substitution degrees are characterized by zeta potential values close to 0 mV, which further denotes an increased agglomeration tendency.

### 2.5. Scanning Electron Microscopy (SEM); Energy Dispersive X-ray Spectroscopy (EDX)

**Figure 4:**
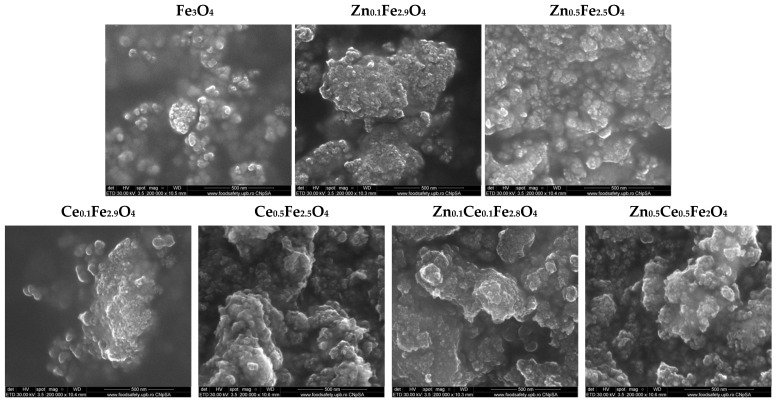
SEM images of the zinc/cerium-substituted magnetite nanoparticles (×200,000).

**Figure 5:**
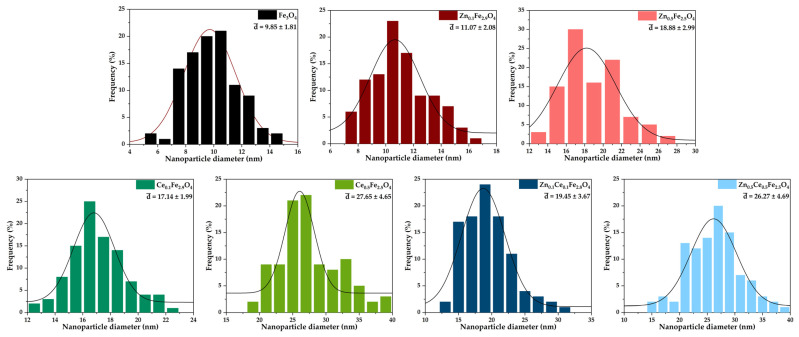
Size distribution of the zinc/cerium-substituted magnetite nanoparticles.

**Figure 6:**
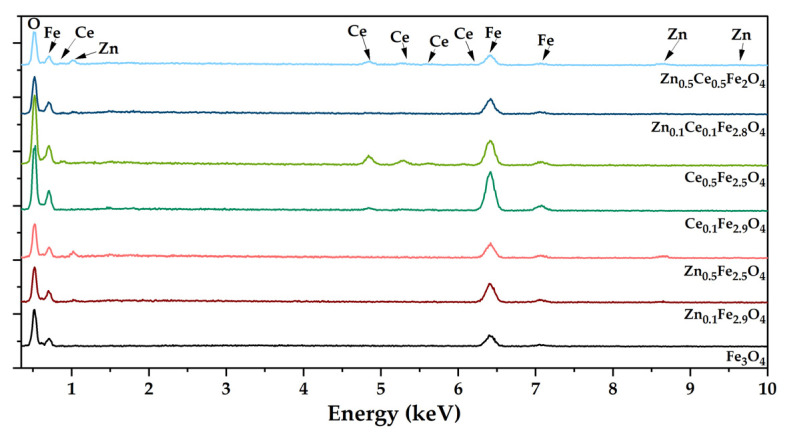
EDX spectra of the zinc/cerium-substituted magnetite nanoparticles.

The morpho-structural properties of the nanostructured systems were investigated through SEM. Micrographs reveal an increased agglomeration tendency and a quasi-spherical morphology within all the obtained nanoparticles (Figure 4). Furthermore, the obtained micrographs were utilized for assessing the size distribution of the nanoparticles using the ImageJ software (version 1.8.0) to measure 100 nanoparticles within each sample (Figure 5). The histograms demonstrate monomodal size distributions for all types of nanoparticles, with average sizes increasing proportionally with the substitution degree. Furthermore, one can deduce that the addition of cerium within the nanostructured systems leads to the formation of larger nanoparticles than the zinc-substituted ones, with the largest nanoparticles being registered for the nanoparticles containing both types of ions. The EDX semi-quantitative measurements demonstrate a proportional increase in the zinc/cerium amount with the substitution degree and a decrease in the iron amount (Figure 6).

### 2.6. Antioxidant Properties

**Figure 7:**
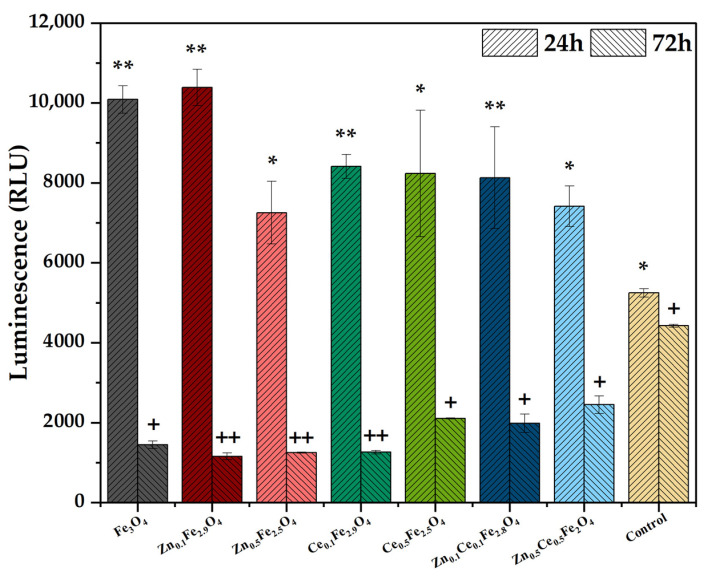
Oxidative stress results for the zinc/cerium-substituted magnetite nanoparticles (BHK cell line; values are expressed as mean ± SD, n = 2; different signs indicate significant differences between the control and each sample; * and +—lower significance; ** and ++—higher significance).

**Figure 8:**
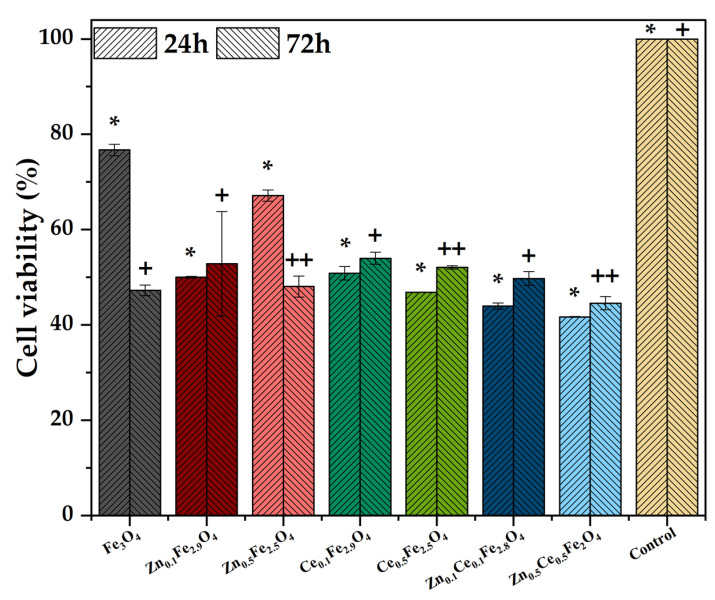
Cell viability results for the zinc/cerium-substituted magnetite nanoparticles (HepG2 cell line; values are expressed as mean ± SD, n = 2; different signs indicate significant differences between the control and each sample; * and +—lower significance; ++—higher significance).

After the physicochemical characterization, the zinc/cerium-substituted nanostructures were biologically evaluated through in vitro ROS-Glo H_2_O_2_ (Figure 7) and MTT (Figure 8) assays in order to assess their antioxidant and anticancer effects, respectively. As it can be observed, the 24-h timepoint witnesses increased levels of oxidative stress within the BHK cells, which is generally caused by an initial shock suffered by the cells after the introduction of a novel compound into the culture media [47]. However, the 72-h mark shows a decrease by more than a half for almost all types of nanoparticles. The most significant reductions were registered for the zinc-containing nanostructures, with no differences between the two concentrations used for substitution, and for the cerium-containing nanoparticles substituted at the lower concentration. Increasing the amount of cerium added leads to higher oxidative stress levels, which is also the case for the double substitutions.

### 2.7. Antitumoral Properties

Furthermore, the MTT assay was employed for assessing the anticancer potential of the substituted nanostructures. It is showcased that cell viability of the HepG2 line is decreased by half in almost all cases, both at 24 and 72-h time intervals. However, there is no significant difference between the control and the pristine or zinc/cerium-substituted magnetite nanoparticles after 24 h. Still, the 72-h timepoint demonstrates significant differences between the control and the samples containing zinc and/or cerium at higher concentrations, denoting differentiated anticancer potential for both zinc and cerium oxides, along with zinc and cerium ions resulted from their release.

### 2.8. Antimicrobial Properties

The subsequent evaluation employed involved assessing the antimicrobial properties of the zinc/cerium-substituted magnetite nanoparticles. Regarding the inhibition zone (Table 4), it seems that the most efficient nanostructures are the ones containing lower amounts of the substitution agents, possibly due to a more facile ion dissolution and, consequently, an enhanced antibacterial effect. Specifically, zinc ions are the most efficient towards Gram-negative bacteria, cerium ions, and against fungi, and Gram-positive strains are the most efficiently inhibited by the presence of both zinc and cerium ions. Subsequently, the MIC values (Table 5) show higher concentrations necessary for inhibiting the growth of *C. albicans* for all types of nanoparticles. Similarly, the *P. aeruginosa* strain is more sensitive towards the action of both zinc ions and zinc oxide. In the case of *S. aureus*, a higher concentration of the nanoparticles containing higher amounts of both substitution agents is necessary.

## 3. Discussion

The current study targeted the development and evaluation of zinc- and/or cerium-substituted magnetite nanoparticles for potential biomedical applications. As results have demonstrated, lower substitution degrees only lead to the replacement of the iron ions with the substitution agents, while higher degrees cause the formation of secondary phases of zinc and cerium oxide. In this context, the present study investigated the effects of both zinc/cerium-substituted magnetite nanoparticles and the synergistic activity of the substituted nanoparticles, as well as the associated secondary oxides.

In regard to the generation of reactive oxygen species, all types of nanoparticles managed to significantly reduce the oxidative stress within the BHK cells. However, it was demonstrated that the presence of cerium oxide slightly reduces the antioxidant properties of the nanostructures. This behavior is in accordance with the previously published literature, where the depletion of intracellular glutathione and increased levels of reactive oxygen species, lipid peroxidation, and superoxide dismutase were reported after cell treatment with cerium oxide nanoparticles [48,49,50]. By contrast, numerous studies demonstrated the antioxidant properties of zinc oxide by scavenging 2,2-diphenyl-1-picrylhydrazyl, 2,2’-azino-bis; 3-ethylbenzothiazoline-6-sulphonic acid, hydroxyl, and superoxide-free radicals [51,52,53,54].

Furthermore, the MTT assay showed that the most pronounced anticancer activity was registered for the nanostructures with the higher substitution degrees. In the case of zinc, results confirm previously published papers reporting cytotoxicity levels dependent upon the content of zinc added due to the formation of zinc oxide [55,56]. Moreover, studies have demonstrated different cytotoxic effects on normal and cancerous cells, with a cell viability decreasing to 80% for the BJ—human foreskin fibroblast cell line, as compared to 50% for the A549—human pulmonary cancer cells [57]. The cerium-substituted nanoparticles behave similarly, with a lower cell viability registered for the Ce_0.5_Fe_2.5_O_4_. While to the best of our knowledge there is no available literature investigating the anticancer properties of cerium-substituted magnetite nanoparticles, cerium oxide nanoparticles are known to normalize the tumor microenvironment through the redox switching mediation between Ce^3+^ and Ce^4+^ [58]. Such as in the case of zinc oxide, previously published results demonstrate increased levels of reactive oxygen species, and induced apoptosis in cancerous WEHI164 cell line and low toxicity levels in normal cells, i.e., L929 line [28]. The differences in cell viability could be explained by the enhanced permeability and retention (EPR) effect and the selective cytotoxicity towards cancer cells [28,59]. In the context of the present study, the antitumoral activity is owed to the synergistic effect between zinc/cerium oxide and the dissolution of zinc and cerium ions.

Hence, the suitability of zinc/cerium-substituted magnetite nanoparticles for anticancer applications was demonstrated both through the antioxidant properties proven towards normal cells and through the antitumoral effects exhibited upon the treatment of cancer cells.

In terms of antimicrobial activity, the available literature demonstrates the potential of both zinc/cerium-substituted magnetite nanoparticles and zinc/cerium oxide nanoparticles against a wide variety of microbial species, e.g., *B. cereus* [19], *B. subtilis* [24,60], *S. aureus* [20,32,60,61,62], *S. enterica* [63], *E. coli* [19,20,24,60,61,62,63,64], *P. aeruginosa* [60,65], *C. jejuni* [63], and *C. albicans* [19]. While the mechanistic pathway behind the activity of zinc/cerium-substituted magnetite nanoparticles has not been precisely determined, it could be assumed that it is based on the targeting of the microbial cell wall, followed by the release of the zinc and cerium ions within the microbial environment and the consequent generation of intracellular reactive oxygen species [31,60]. Considering the results of this study, the improved antimicrobial activity of the nanoparticles obtained at lower substitution degrees could be owed to a more facile dissolution of the zinc and cerium ions compared to the associated oxides. Moreover, the nanoparticle size could also play a key role in inhibiting the microbial growth [61], since higher substitution degrees resulted in the increase in the average nanoparticle size, as shown in SEM images.

Therefore, the present study successfully demonstrated the potential of zinc and cerium substitutions for tuning the antioxidant, antitumoral, and antimicrobial properties of magnetite nanoparticles. In this manner, zinc/cerium-substituted magnetite nanoparticles could act as strong candidates for drug delivery systems in a plethora of biomedical applications, such as cancer treatment, wound dressings, or implant coatings.

## 4. Materials and Methods

### 4.1. Materials

Ferric chloride hexahydrate (FeCl_3_·6H_2_O), ferrous sulphate heptahydrate (FeSO_4_·7H_2_O), zinc chloride (ZnCl_2_), cerium (III) nitrate hexahydrate (Ce(NO_3_)_3_·6H_2_O), and sodium hydroxide (NaOH) were purchased from Sigma-Aldrich Merck (Darmstadt, Germany) and used as such.

For the ICP-MS analysis, calibration solutions were prepared from multi-element calibration standards purchased from Agilent Technologies (Santa Clara, CA, USA, p.n: 8500-6944 and 8500-6940). The sample preparation was conducted using trace analysis grade nitric acid (HNO_3_) purchased from Honeywell (Charlotte, NC, USA), while all dilutions were performed with Milli-Q water (18 MΩ·cm). 

The Baby Hamster Kidney fibroblasts (BHK) and human hepatocarcinoma (HepG2) cell lines were provided by the Institute for Diagnosis and Animal Health (I.D.A.H.).

The microbial strains (*S. aureus* ATCC 25923, *P. aeruginosa* ATCC 15442, and *C. albicans* ATCC 10231) used for the antimicrobial assays were provided by the Microbiology and Immunology Department from the Faculty of Biology, University of Bucharest.

### 4.2. Synthesis of Zinc/Cerium-Substituted Magnetite Nanoparticles

All nanoparticle samples were obtained through the co-precipitation method [11,66]. The iron ion precursors were dissolved in ultrapure water in a molar ratio of Fe^2+^:Fe^3+^ = 1:2. For the zinc and/or cerium-substituted nanoparticles, the zinc and/or cerium precursors were dissolved in the same solutions in appropriate amounts. The obtained solutions were dripped into the 1M NaOH solution using a peristaltic pump, which allowed for the precipitation of the zinc/cerium-substituted magnetite nanoparticles. Subsequently, the precipitate was separated by applying a high-power NdFeB magnet under the reaction beaker and afterward washed with deionized water until pH = 7. Table 6 summarizes all the obtained nanoparticle samples.

### 4.3. Morpho-Structural Characterization

#### 4.3.1. X-ray Diffraction (XRD) Coupled with Rietveld Refinement

The compositional and structural evaluation of the samples was performed through the XRD analysis, using a PANalytical Empyrean diffractometer (PANalytical, Almelo, The Netherlands), equipped with a CuKα radiation source. The patterns were recorded in a Bragg-Brentano configuration, with a 2θ angle varying from 10° to 80°, a step size of 0.0256°, and time per step of 1 s. The Rietveld refinement method was afterward per-formed using the HighScore Plus software (version 3.0.5, PANalytical, Almelo, The Netherlands), to determine the average crystallite size and the unit cell parameters. Diffractogram fittings were considered acceptable if the goodness of fit < 4.

#### 4.3.2. Inductively Coupled Plasma Mass Spectrometry (ICP-MS)

The content of cerium and zinc ions from the substituted magnetite nanoparticles was evaluated in triplicate with an Agilent 8800 Triple Quadrupole ICP-MS instrument (Agilent Technologies, Tokyo, Japan). Both elements were quantified using the calibration curve method. The instrumental parameters were set to 1550 W radiofrequency power, 1 L/min of argon gas as carrier, 0.7 mL/min of helium, and 0.1 rpm for the peristaltic pump. The method was linear in the range of 1–25 µg/L, with correlation coefficients of 0.9992, 0.9997, and 0.9991 for iron, cerium, and zinc, respectively.

Using an Ethos UP microwave system (Milestone Inc., Sorisole, Italy), approximately 10 mg of each sample powder was weighed in special TFM digestion vessels and further digested in 8 mL of concentrated HNO_3_. The digested solutions were transferred quantitatively to 50-mL volumetric flasks and filled to capacity with ultrapure water. Subsequently, a 1000-fold dilution was performed to ensure that the actual values of the samples are within the calibration range of the instrument. The measured concentrations were achieved for isotopes ^56^Fe, ^66^Zn, and ^140^Ce, and are expressed in µg/L. Finally, the final concentrations for each element are expressed in µg/mg, being calculated by applying the dilution factors used in the sample preparation step and the exact mass of the sample taken into analysis.

#### 4.3.3. X-ray Photoelectron Spectroscopy (XPS)

The analysis of the material’s surface chemistry was performed on an ASPECT pho-toelectron spectrometer (Scienta Omicron Technology GmbH, Taunusstein, Germany), equipped with a 160-mm hemispherical energy analyzer with a 1D detector. All measurements were made in a vacuum (~2 × 10^−9^ mbar), employing a polychromatic Mg Kα X-ray source at 1253.6 eV with a power of 300 W. The diameter of the analysis spot was 1.3 mm. The XPS survey wide scan spectra were recorded from −5 to 1200 eV (binding energy), with a pass energy of 200 eV, while the high-resolution spectra were recorded with a pass energy of 20 eV and step of 0.1 eV. The obtained XPS spectra were post-processed to eliminate satellite peaks and evaluated with CasaXPS software (version 2.3.25). For the determination of background lines, the Shirley fitting function was used. The binding energy scale was calibrated based on the C-C component in the C 1s signal (by default at 248.8 eV) associated with the adventitious carbon. 

#### 4.3.4. Dynamic Light Scattering (DLS) and Zeta Potential

The particle size distribution and particle zeta potential were determined in triplicate with a DelsaMax Pro equipment (Backman Coulter, Brea, CA, USA). The nanoparticles were first dispersed in deionized water (0.3 mg/mL, pH~6.9), then homogenized for 10 min using an ultrasonic bath. Subsequently, a small amount was introduced inside the measurement cell and each sample was measured in triplicate.

#### 4.3.5. Scanning Electron Microscopy (SEM): Energy Dispersive X-ray Spectroscopy (EDX)

The morphological and elemental aspects of the samples were investigated using the Inspect F50 scanning electron microscope (Thermo Fisher, Eindhoven, The Netherlands), equipped with an energy dispersive spectrometer. SEM images were acquired at different magnifications using the Everhart–Thornley secondary electrons detector and an accelerating voltage of 30 KeV energy.

### 4.4. Biological Evaluations

#### 4.4.1. ROS-Glo H_2_O_2_ Assay

The evaluation of oxidative stress induced by the zinc/cerium-substituted nanoparticles on the BHK cell line was made through the ROS-Glo assay (Promega, Madison, WI, United States) [1]. A total of 10 mg of each sample was dispersed in 200 µL cell suspension, followed by the addition of the cell medium until a final concentration of 5 mg/mL. The H_2_O_2_ levels were measured after 24 and 72 h of incubation based on the manufacturer instructions with a SpectraMax i3x Multi-Mode microplate reader. The results were collected with the Soft-Max Pro 6 software and presented as relative light units (RLU).

#### 4.4.2. MTT Assay

The cell viability was determined through the MTT assay on a HepG2 cell line [1]. All powders were UV-sterilized for 1 h. A total of 10 mg of each sample was dispersed in 200 µL cell suspension, followed by the addition of the cell medium until a final concentration of 5 mg/mL, and maintained for 24 h and 72 h. After removing the media, the cells were thoroughly washed with Phosphate-buffered saline (PBS) and incubated at 37 °C and 5% CO_2_ in the absence of light for 2 h with a (3-(4,5-dimethylthiazol-2-yl)-2,5-diphenyltetrazolium bromide (MTT) solution. After reduction of the MTT and removal of the resulting solution, the precipitated formazan crystals were solubilized in isopropanol. The optical density (OD) was spectrophotometrically measured at 595 nm using a SpectraMax i3x Mul-ti-Mode microplate reader. The results were collected with the SoftMax Pro 6 software, and the cell viability of the samples was calculated using Equation (1):

(1) $$\mathrm{Cell}\;\mathrm{viability}\left(\%\right)=\frac{\mathrm{OD}595\;\mathrm{sample}}{\mathrm{OD}595\;\mathrm{control}}\;\times \;100$$

### 4.5. Antimicrobial Activity

The antimicrobial protocols were performed according to previously published studies [47,67,68]. The antimicrobial activity was assessed for all pristine and zinc/cerium-substituted nanoparticles after sterilization with UV radiation for 20 min. The nanoparticles were dispersed in sterile deionized water at a concentration of 1 mg/mL to obtain a stock solution. All measurements were performed in duplicate.

The inhibition zone diameter (qualitative antimicrobial assessment) was determined based on the adapted diffusion test from the Clinical & Laboratory Standards Institute (CLSI) guidelines. In brief, a microbial suspension of 1–3 × 10^8^ CFU/mL (equivalent of 0.5 McFarland density standard) was swab-inoculated in Petri dishes containing the Mueller–Hinton agar medium for *S. aureus* and *P. aeruginosa* bacteria and Sabouraud Dextrose broth for *C. albicans* yeast. Stock solutions of the obtained nanoparticles were added dropwise (10 µL) on the inoculated plates and incubated at 37 °C for 24 h. The resulting inhibition zone diameter was measured, and the results were expressed in mm.

The minimum inhibitory concentration—MIC (quantitative antimicrobial assessment) was determined using the microdilution technique in 96-well plates. Specifically, each of the tested nanoparticles were consecutively diluted from 2 mg/mL to 0.015625 mg/mL in each type of nutritive broth and a microbial suspension of ~10^6^ CFU/mL was utilized to inoculate the plates. The cultures were incubated at 37 °C for 24 h. The MIC was assessed by a naked-eye analysis, where the lowest concentration of the nanoparticles that inhibited the visible growth of the microbial strains was considered [69].

### 4.6. Statistical Analysis

The biological evaluations were performed in duplicate, and the data are represented as mean ± standard deviation. The obtaining results were statistically analyzed using the one-way analysis of variance (ANOVA) followed by the two-tailed *t*-test with Bonferroni post hoc correction (*p* < 0.05). Data comparison was made with the GraphPad Prism 9 software (San Diego, CA, USA).

## 5. Conclusions

The hypothesis behind the design of the current study was based on the possibility of enhancing the properties of magnetite nanoparticles by incorporating zinc and/or cerium ions within its crystal lattice. The physicochemical characterization of the obtained nanostructures revealed the successful substitution of the iron ions with zinc and/or cerium by modifications of the magnetite unit cell, as well as the formation of zinc and/or cerium oxides as secondary mineral phases at higher substitution degrees. Furthermore, the substitution process resulted in the increase in the average nanoparticle size and, consequently, in more stable nanoparticles in terms of the agglomeration tendency. The biological evaluation confirmed the antioxidant, anticancer, and antimicrobial properties of the nanostructures, with the efficiency dependent upon the type and concentration of the ions used for the substitution. To summarize, as demonstrated through this study, the substitution of magnetite nanoparticles with zinc/cerium ions represents a promising approach towards the development of biomedical applications.

## Figures and Tables

**Table 1:** Unit cell parameters, average crystallite size, and crystallinity acquired from the Rietveld refinement of the diffractograms.

Sample		Unit Cell Parameters						Average Crystallite Size ± Standard Deviation (SD) [nm]	Crystallinity [%]
		a [Å]	b [Å]	c [Å]	α [°]	β [°]	γ [°]		
Fe_3_O_4_		8.37	8.37	8.37	90	90	90	3.20 ± 0.13	16.72
Zn_0.1_Fe_2.9_O_4_		8.38	8.38	8.38	90	90	90	4.05 ± 0.23	13.72
Zn_0.5_Fe_2.5_O_4_	Fe_3_O_4_ 77.9%	8.43	8.43	8.43	90	90	90	2.09 ± 0.20	20.57
	ZnO 22.1%	3.56	3.56	5.23	90	90	120	1.94 ± 0.06	
Ce_0.1_Fe_2.9_O_4_		8.39	8.39	8.39	90	90	90	4.32 ± 0.16	10.48
Ce_0.5_Fe_2.5_O_4_	Fe_3_O_4_ 73.6%	8.36	8.36	8.36	90	90	90	9.55 ± 0.81	10.88
	Ce_2_O_3_ 26.4%	3.83	3.83	6.45	90	90	120	2.50 ± 0.09	
Zn_0.1_Ce_0.1_Fe_2.8_O_4_		8.39	8.39	8.39	90	90	90	5.49 ± 0.44	10.26
Zn_0.5_Ce_0.5_Fe_2_O_4_	Fe_3_O_4_ 72.4%	8.42	8.42	8.42	90	90	90	8.93 ± 0.60	11.24
	ZnO 10.0%	3.63	3.63	5.23	90	90	120	3.87 ± 1.03	
	Ce_2_O_3_ 17.6%	3.79	3.79	6.35	90	90	120	2.87 ± 0.17	

**Table 2:** Experimental ICP-MS results and calculated values obtained for Fe, Zn, and Ce content (± SD, n = 3).

**Element**	**Calculated Concentration (µg/mg)**						
	**Fe_3_O_4_**	**Zn_0.1_Fe_2.9_O_4_**	**Zn_0.5_Fe_2.5_O_4_**	**Ce_0.1_Fe_2.9_O_4_**	**Ce_0.5_Fe_2.5_O_4_**	**Zn_0.1_Ce_0.1_Fe_2.8_O_4_**	**Zn_0.5_Ce_0.5_Fe_2_O_4_**
^56^Fe	724.14	697.3	591.97	675.54	510.95	649.81	402.15
^66^Zn	-	27.9	137.42	-	-	26.94	116.7
^140^Ce	-	-	-	58.24	255.47	58.02	251.35
**Element**	**Experimental Concentration (µg/mg)**						
	**Fe_3_O_4_**	**Zn_0.1_Fe_2.9_O_4_**	**Zn_0.5_Fe_2.5_O_4_**	**Ce_0.1_Fe_2.9_O_4_**	**Ce_0.5_Fe_2.5_O_4_**	**Zn_0.1_Ce_0.1_Fe_2.8_O_4_**	**Zn_0.5_Ce_0.5_Fe_2_O_4_**
^56^Fe	438 ± 61	405 ± 57	349 ± 51	406 ± 58	310 ± 45	399 ± 56	264 ± 38
^66^Zn	<LoQ *	18.5 ± 1.4	93.7 ± 8.4	-	-	18.04 ± 1.3	85 ± 7.4
^140^Ce	<LoQ *	-	-	64.9 ± 1.2	348.5 ± 12.7	64.3 ± 0.6	296.8 ± 10.9

* Concentration is below limit of quantification. The instrument signal (cps) was in the range of 1499 ± 20 cps, similar to a blank sample.

**Table 3:** Theoretical and experimentally estimated stoichiometry of the zinc/cerium-substituted nanoparticles.

Sample	Theoretical Stoichiometry	Estimated Stoichiometry
Zn_0.1_Fe_2.9_O_4_	Zn:Fe = 0.1:2.9	Zn:Fe = 0.1:2.9
Zn_0.5_Fe_2.5_O_4_	Zn:Fe = 0.5:2.5	Zn:Fe = 0.36:2.64
Ce_0.1_Fe_2.9_O_4_	Ce:Fe = 0.1:2.9	Ce:Fe = 0.16:2.84
Ce_0.5_Fe_2.5_O_4_	Ce:Fe = 0.5:2.5	Ce:Fe = 0.59:2.41
Zn_0.1_Ce_0.1_Fe_2.8_O_4_	Zn:Ce:Fe = 0.1:0.1:2.8	Zn:Ce:Fe = 0.1:0.16:2.74
Zn_0.5_Ce_0.5_Fe_2_O_4_	Zn:Ce:Fe = 0.5:0.5:2	Zn:Ce:Fe = 0.31:0.51:2.18

**Table 4:** Inhibition zone diameter measured for the zinc/cerium-substituted magnetite nanoparticles (average values—n = 2).

Microbial Strain	Inhibition Zone Diameter (mm)						
	Fe_3_O_4_	Zn_0.1_Fe_2.9_O_4_	Zn_0.5_Fe_2.5_O_4_	Ce_0.1_Fe_2.9_O_4_	Ce_0.5_Fe_2.5_O_4_	Zn_0.1_Ce_0.1_Fe_3_O_4_	Zn_0.5_Ce_0.5_Fe_3_O_4_
*S. aureus*	5	5	4	5	5	6	5
*P. aeruginosa*	5	6	4	5	5	4	5
*C. albicans*	5	5	4	6	5	4	4

**Table 5:** MIC values determined for the zinc/cerium-substituted magnetite nanoparticles (average values—n = 2).

Microbial Strain	MIC (mg/mL)						
	Fe_3_O_4_	Zn_0.1_Fe_2.9_O_4_	Zn_0.5_Fe_2.5_O_4_	Ce_0.1_Fe_2.9_O_4_	Ce_0.5_Fe_2.5_O_4_	Zn_0.1_Ce_0.1_Fe_3_O_4_	Zn_0.5_Ce_0.5_Fe_3_O_4_
*S. aureus*	1	1	1	1	1	1	2
*P. aeruginosa*	1	1	1	1	2	2	2
*C. albicans*	2	2	2	2	2	2	2

**Table 6:** Summary of the nanoparticle samples obtained.

Sample Code	Degree of Substitution with Zinc (Molar%)	Degree of Substitution with Cerium (Molar%)
Fe_3_O_4_	-	-
Zn_0.1_Fe_2.9_O_4_	3.33	-
Zn_0.5_Fe_2.5_O_4_	16.66	-
Ce_0.1_Fe_2.9_O_4_	-	3.33
Ce_0.5_Fe_2.5_O_4_	-	16.66
Zn_0.1_Ce_0.1_Fe_2.8_O_4_	3.33	3.33
Zn_0.5_Ce_0.5_Fe_2_O_4_	16.66	16.66

## Data Availability

No new data were created.
